# BIRI: a new approach for automatically discovering and indexing available public bioinformatics resources from the literature

**DOI:** 10.1186/1471-2105-10-320

**Published:** 2009-10-07

**Authors:** Guillermo de la Calle, Miguel García-Remesal, Stefano Chiesa, Diana de la Iglesia, Victor Maojo

**Affiliations:** 1 Dept Inteligencia Artificial, Facultad de Informática, Universidad Politécnica de Madrid, Campus de Montegancedo S/N, 28660 Boadilla del Monte, Madrid, Spain

## Abstract

**Background:**

The rapid evolution of Internet technologies and the collaborative approaches that dominate the field have stimulated the development of numerous bioinformatics resources. To address this new framework, several initiatives have tried to organize these services and resources. In this paper, we present the BioInformatics Resource Inventory (BIRI), a new approach for automatically discovering and indexing available public bioinformatics resources using information extracted from the scientific literature. The index generated can be automatically updated by adding additional manuscripts describing new resources. We have developed web services and applications to test and validate our approach. It has not been designed to replace current indexes but to extend their capabilities with richer functionalities.

**Results:**

We developed a web service to provide a set of high-level query primitives to access the index. The web service can be used by third-party web services or web-based applications. To test the web service, we created a pilot web application to access a preliminary knowledge base of resources. We tested our tool using an initial set of 400 abstracts. Almost 90% of the resources described in the abstracts were correctly classified. More than 500 descriptions of functionalities were extracted.

**Conclusion:**

These experiments suggest the feasibility of our approach for automatically discovering and indexing current and future bioinformatics resources. Given the domain-independent characteristics of this tool, it is currently being applied by the authors in other areas, such as medical nanoinformatics. BIRI is available at .

## Background

The number of public online bioinformatics resources has grown exponentially over the past few years. Bioinformatics professionals can access and use a large number of resources for their research --including databases, tools and services. Discovering and accessing the appropriate bioinformatics resource for a specific research task has become increasingly important, as suggested in earlier reports [1].

To address this issue, various significant projects and initiatives have been carried out, leading to several pioneering indexes of bioinformatics resources that are currently available over the Internet. For instance, BioPortal [2] is a web repository of biomedical ontology resources, developed at the National Center for Biomedical Ontology (NCBO) [3]. This application enables collaborative development of biomedical ontologies. BioPortal includes a service called Open Biomedical Resources (OBR) for annotating and indexing biomedical resources [4]. Resources are annotated using concepts from a domain ontology. The OBR service enables researchers to locate resources by specifying ontology concepts.

Other examples of such indexes include the biomedical database collection compiled by Galperin [5]--a yearly updated web-based list of molecular biology databases sorted in alphabetical order-- or the Bioinformatics Links Directory (BLD) [6,7] --a catalogue of links to bioinformatics resources, tools and databases classified into eleven major categories-- where resources can be searched using keyword-based queries.

The European Bioinformatics Institute (EBI) provides a searchable index of an alphabetically-sorted inventory of bioinformatics resources [8]. Resources are classified according to the type of service they provide--databases, tools and (web) services. The index includes both internal and external resources.

The BioMoby platform provides an annotated registry of bioinformatics web services enabling other applications to integrate and use such services [9,10]. There are various major installations of BioMoby, such as, for instance, the PlaNet Consortium [11], the Australian Centre for Plant Functional Genomics [12], the Generation Challenge Program of the Consultative Group for International Agricultural Research [13], Genome Canada [14], or the Spanish National Institute of Bioinformatics (INB) [15]. They provide access to different bioinformatics resources depending on their own interests and needs.

A consortium composed of the seven US National Centers for Biomedical Computing [16] has recently developed another index of bioinformatics resources called iTools [17,18]. Web services are used here to provide access to resources that are annotated according to their functionality. A web-based interface enables researchers to locate the resources they need using advanced search and visual navigation tools. Another initiative for organizing bioinformatics resources is the BIO2RDF system [19]. This system considers the information contained in biological databases, providing uniform access to biological data stored in public databases. Data is converted into RDF format using a common reference ontology. More than 20 publicly available databases --e.g. Entrez Gene, OMIM, GO, OBO, PDB, GeneBank, Prosite, etc. -- have been successfully integrated, including more than 160 million RDF documents.

In this paper we present a tool to automatically create a searchable index of bioinformatics resources from the scientific literature. The resources are annotated with metadata regarding their functionality. Metadata are extracted from abstracts of research papers retrieved from PubMed^® ^[20] and the ISI Web of Knowledge^® ^[21] using pattern-matching techniques. The generated index can be incrementally updated by feeding our tool with abstracts of articles describing new resources. In the evaluation section, we present and analyze some figures that compare the results of our approach with other existing indexes.

## Methods

We extract relevant information about bioinformatics resources from papers published in high-impact journals. We focus on manuscript abstracts, which tend to condense the key information. We use classic natural language processing techniques--such as, for instance, tokenizers, parsers, transition networks or part-of-speech taggers--to extract this information. These techniques provide the basic framework for extracting data from textual resources [22]. All the techniques we have used are detailed in the following sections. The outcome of this process is an indexed knowledge base including the most relevant information about the resources.

### Building the knowledge base

**Figure 1:**
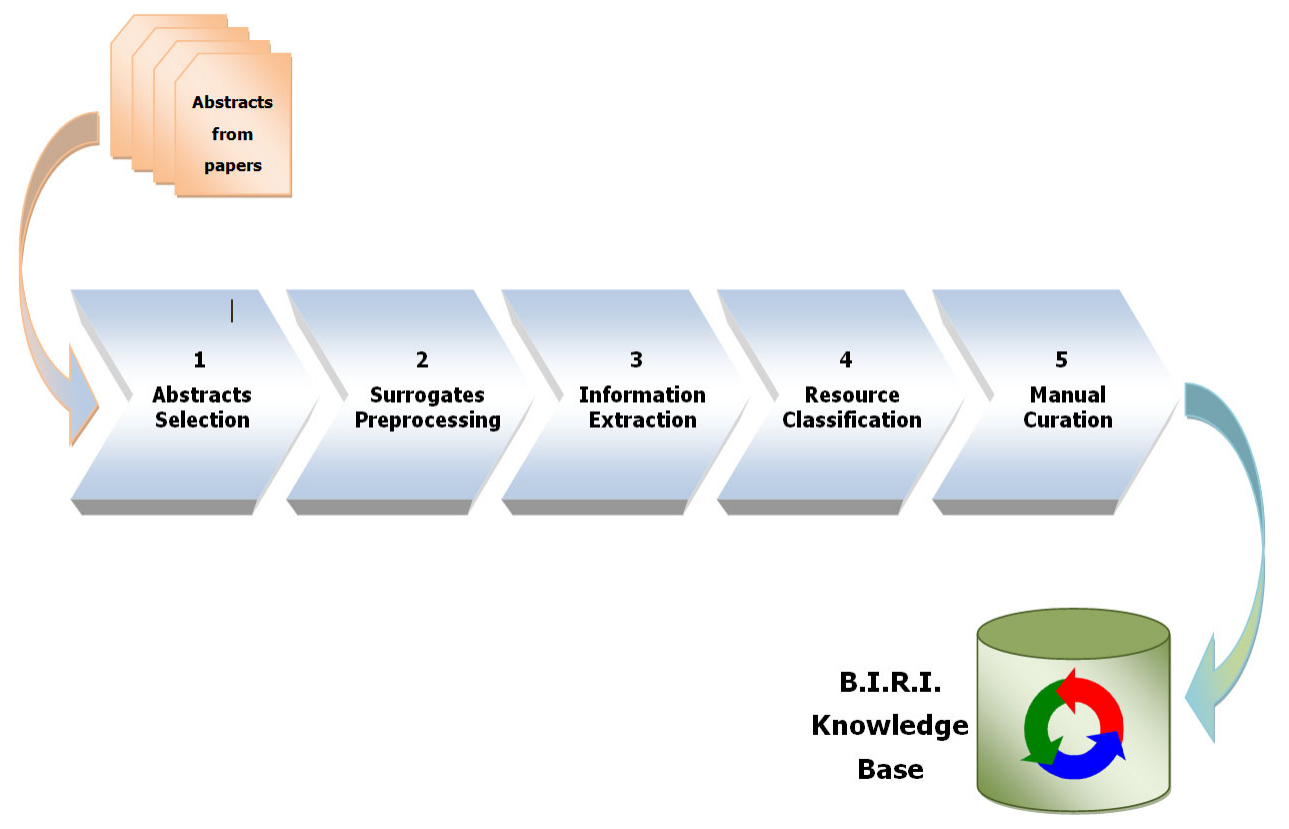
**BIRI knowledge base automatic construction phases**. We propose a five-step methodology for automatically building the knowledge base: i) abstract selection, ii) surrogate pre-processing, iii) information extraction, iv) resource classification and v) manual curation. Abstracts are extracted from scientific papers taken from the ISI Web of Knowledge^®^.

We propose a five-phase method for automatically generating a knowledge base of bioinformatics resources using research paper abstracts. Figure 1 shows an overview of the proposed methodology. Next we detail the five activities.

#### Abstract selection and generation of abstract surrogates

First we retrieve a collection of papers from well-known scientific literature web sites. Each paper is then analyzed to produce a structured surrogate, including the title, authors, abstract and electronic references to the paper--i.e. the PubMed^® ^Identifier (PMID). The structure of the surrogate and a complete example is shown within the "Papers" section in Table 1. Such surrogates simplify the pattern-matching process. For instance, the title of the paper usually contains the name of the resources. We use that knowledge as a heuristic to discard unnecessary searches in other sections of the manuscripts.

**Table 1:** Complete resource information stored in the knowledge base

**RESOURCE**			
**Name:**	GenePublisher		

**FUNCTIONALITIES**			

**Functionality 1**: analysis of DNA microarray data			

**Category:**	analysis	**Domain:**	DNA

**Category:**	analysis	**Domain:**	Microarray

**Input:**		**Output:**	dna microarray data

**PAPERS**			

**Paper 1**: GenePublisher: automated analysis of DNA microarray data.			

**Authors:**	Knudsen, S; Workman, C; Sicheritz-Ponten, T; Friis, C		

**Abstract:**	GenePublisher, a system for automatic analysis of data from DNA microarray experiments, has been implemented with a web interface at . Raw data are uploaded to the server together with a specification of the data. The server performs normalization, statistical analysis and visualization of the data. The results are run against databases of signal transduction pathways, metabolic pathways and promoter sequences in order to extract more information. The results of the entire analysis are summarized in report form and returned to the user.		

**PMID:**	12824347	**ISIID:**	000183832900039

The table shows an example of all the information about resources in the BIRI Knowledge Base. For each resource, the system stores the information that it has extracted from the literature. Additional links, if any, are provided to the resource's official web page and to the PubMed^® ^website.

#### Surrogate pre-processing

Once the surrogates have been generated, the titles and abstracts are divided into sentences, using strong punctuation marks as sentence delimiters. Then, each sentence is pre-processed by a lexical analyzer. The analyzer extracts the words of the sentences using blanks and punctuation marks as word delimiters. This produces a sorted set of tokens tagged with lexical annotations. Next, each token is labelled with its corresponding part-of-speech (POS) tag using a probabilistic POS-tagger [23]. Stop words--such as articles, prepositions, etc.--are also filtered during this process. Finally, a stemming activity is performed to reduce each token to its root form, thus returning a set of lexemes tagged with morphological information [24].

#### Manual generation of patterns and transition networks

To automatically extract information from the abstracts, a group of five experts with background in different bioinformatics and text mining techniques created a set of linguistic patterns and then analyzed the selected abstracts. The set of linguistic patterns characterizes the type of information to be extracted. We used an initial training set of 100 abstracts of randomly chosen papers from PubMed^®^, describing bioinformatics resources--e.g. database and software papers published in the BMC Bioinformatics Journal [25] or application notes from the Bioinformatics Journal [26]. The selected abstracts were analyzed to discover and identify three different types of patterns: i) resource-naming patterns (RNP), ii) functionality patterns (FP), and iii) classification patterns (CP). RNPs aim to automatically extract the resource names together with their corresponding URL. Conversely, FPs aim to extract short textual descriptions of resource functionalities. By contrast, CPs focus on either i) the category of the resource or ii) its target domain.

A set of categories and domains was defined by the team of experts based on the taxonomy developed by the Bioinformatics Links Directory. This set includes the key categories of bioinformatics resources--e.g. databases, annotation services, visualization tools, etc. Each category is represented by a preferred name, together with a set of synonyms. Each resource category is linked to a collection of topics for its target domain(s). For instance, examples of domains for the "database" category are "DNA", "microarray", "polymorphisms" or "protein". Table 2 shows the complete list of BIRI categories and domains.

**Table 2:** Full list of BIRI categories and domains

**Categories**					**Domains**	
		
Alignment	Design	Inference	Profiling		DNA	Phylogenetics
Analysis	Discovery	Knowledgebase	Repository		Expression	Polymorphism
Annotation	Evaluation	Mapping	Retrieval		Gene	Protein
Bootstrapping	Exploration	Mining	Search		Genome	RNA
Classification	Footprinting	Pattern-matching	Summary		Microarray	
Comparison	Framework	Prediction	Taxonomy			
Database	Identification	Processing	Visualization			

The table shows the full list of BIRI categories and domains. This list is composed of 28 categories and nine domains. Each domain has an associated set of categories and each category can belong to several domains.

**Figure 2:**
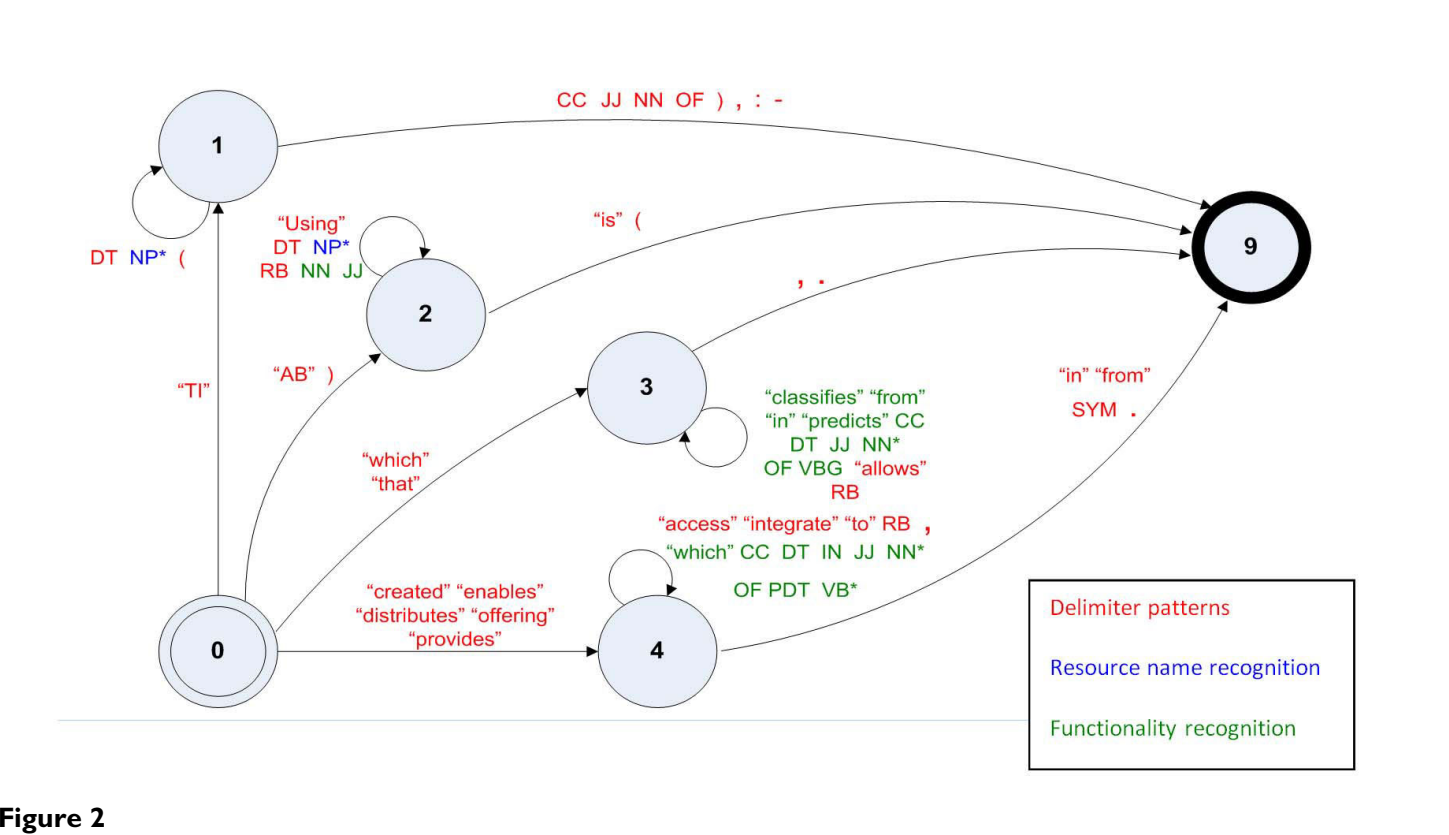
**Fragment of the transition network to detect resource names**. This is an extract of the transition network used to identify resource names from the surrogates. According to the inputs (labels over the arcs), the transition network passes from state 0 to other states until it reaches a final state that determines the identification of a new resource. Different tags are used as labels: punctuation marks and words within quotes represent literals---i.e. marks or words that are in the surrogates---, and abbreviations in capital letters that represent morphological tags. For instance, NP is a proper noun, CC represents a conjunction, NN refers to common noun, DT symbolizes a determiner, JJ denotes an adjective, RB is a general adverb, OF and IN represent prepositions, PDT denotes a determiner, VB represents a verb, VBG refers to a verb in gerund form (ending in -*ing*) or SYM is a symbol or formula.

Once the experts had identified the patterns, they were translated into Transition Networks (TNs) [27]. TNs are simple but effective abstract machines that determine whether a string belongs to a language defined by a regular expression. We adopted this approach since TNs are simple yet powerful tools suitable for performing pattern-matching tasks. In our approach, TNs are used to recognize instances of the patterns in the abstracts. We defined two different TNs. The first TN is designed to detect and extract the names of the resources together with brief descriptions of their functionalities. This TN is based on the extracted RNPs and FPs. Conversely, the second TN is designed to classify the resources into different categories depending on their functionalities and target domains. Figure 2 shows an extract of the first TN. This is represented as a finite state machine. Each state is represented by a numbered circle. The labelled edges show the required textual input for transiting from one state to another. Given an input, the aim is to reach a final state (for instance, state 9 in Figure 2) from an initial state (for example, state 0). If a final state is reached, then the TN outputs the extracted information for the matched resource.

#### Information extraction

Extracted patterns are applied to the abstracts as follows. First, the abstract is analyzed by the first TN to locate the substrings that match any of the patterns. Then, the relevant information is extracted from the substring. For instance, the input sentence "The FSSP database of structurally aligned protein fold families" matches the RNP [*"The" + Proper Noun + "database" + "of"*]. In this case, the analyzer identifies "FSSP" as a proper noun. Another example of RNP could be [*Proper Noun + ":" + "a"*] that would match the sentence "EMBL-Align: a public nucleotide and amino acid multiple sequence database. In this case, "EMBL-Align" would be recognized as a proper noun.

FPs are often more complex to match than RNPs and CPs since their associated patterns require further lexical components for instantiation--e.g. verb tenses, part-of-speech tags, etc. For instance, the input sentence "A novel method for fast and accurate multiple sequence alignment" matches the following pattern [*"A" + JJ*^+^+ "method" + "for" + (JJ + (CC + JJ)?)? + JJ* + NN^+^*+ "."*], where JJ represents an adjective, CC identifies a conjunction and NN is a common name. We use special characters of regular expressions to specify the patterns, where '?' represents optionality, '^+^' one or more occurrences and '*' zero or more occurrences of the component. In the last example, the functionality extracted would be "sequence alignment". During the pattern-matching process, the instantiation of one of the patterns triggers a procedure that extracts and stores the relevant information in the knowledge base.

#### Resources classification

Once the name and the description of the functionalities for a given resource have been extracted, we run the second TN to perform the classification. The TN is fed with the extracted description(s) of the functionality of the current resource, and it tries to match this description with a resource category and domain. If successfully matched, the resource is labelled with the preferred term associated with the respective category or domain. Note that a resource could be assigned to several categories or domains. For instance, one possible pattern for detecting and extracting a resource category and domain is: [("program" | "tool" | "server") + ("for"|"to") + (JJ* + (NN | NNS))? + "^annot" + ("of" + JJ* + (NN | NNS)^+^)*? + "."*]. Applying this pattern to the sentence "AMIGene: a tool for annotation of microbial genes", the category we get is "annotation" and the resource domain is "genes".

Additionally, any extra information--such as resource inputs and outputs--can also be extracted by the second TN when available. We refer to the type of data received by the resources when they are invoked as 'inputs' and to the type of data they return as a result as 'outputs'. For instance, applying the pattern ["discovery" + "of" + (JJ + CC?)* + (NN | NNS)^+^+ ("within" | "in") + JJ* + (NN | NNS)^+^*+ "."*] to the sentence "We present here a new tool for discovery of unstructured or disordered regions within proteins.", "proteins" would be the resource input data.

#### Data curation

Once all relevant information contained in the abstracts has been extracted, a team of experts reviews the contents of the knowledge base to assess its correctness. The curation process mainly focuses on the detection of categories and domains incorrectly assigned to specific resources. Then, the experts compare the previously extracted functionality with the assigned categories and domains. If any errors are detected, the inaccurate entries are removed from the BIRI knowledge base.

### Description of the knowledge base

Once the information has been filtered, it is stored in a knowledge base. For each discovered resource, we record the following data: i) the name of the resource, ii) its corresponding URL, iii) a set of natural language descriptions of its functionalities, iv) the resource's assigned categories, v) its target domain(s) and vi) the resource's inputs and outputs when available. Using the same example as above, this information could be extended and used to automatically orchestrate workflows involving multiple resources. The knowledge base also stores data about the original papers, including the paper's title, author(s), abstract and PubMed^® ^and ISI Web of Knowledge^® ^identifiers.

**Figure 3:**
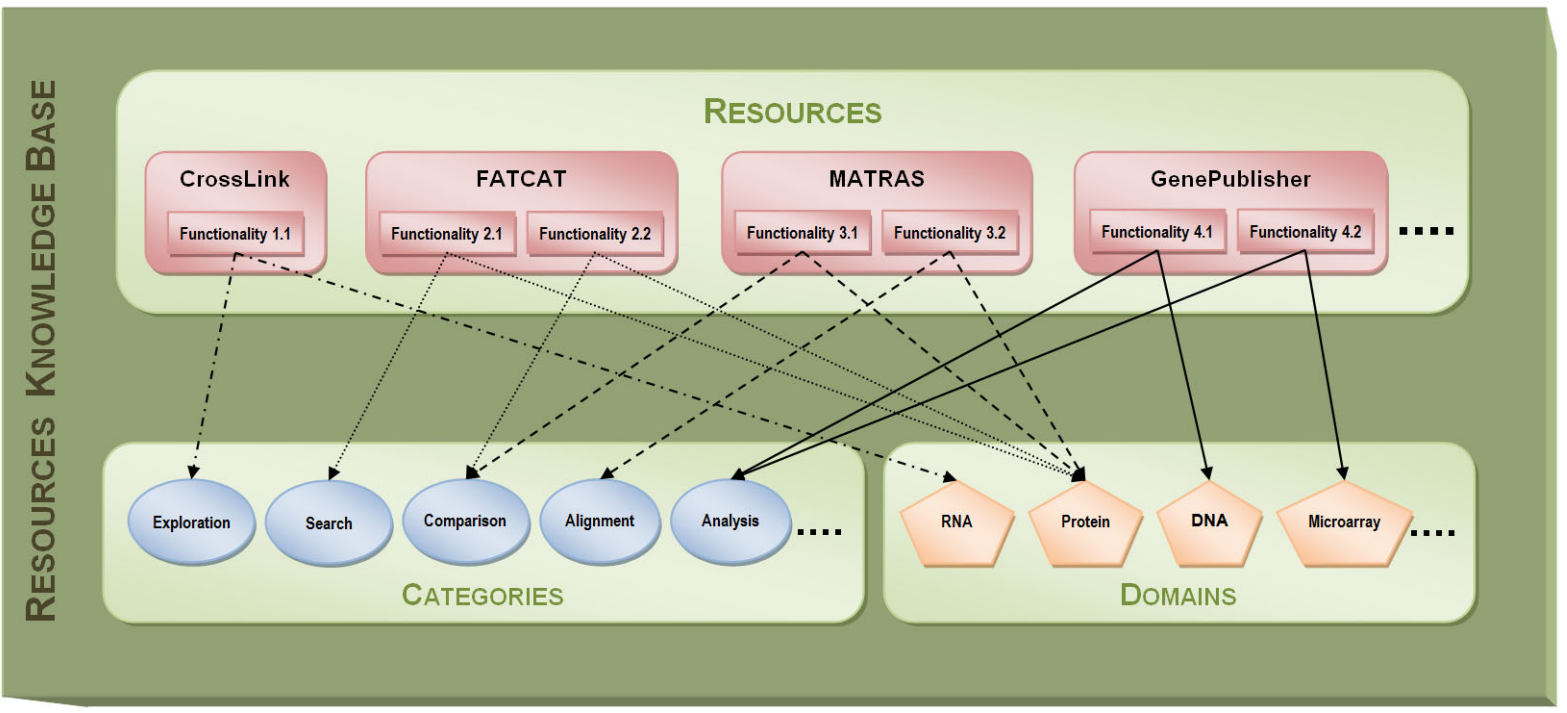
**Conceptual schema of the BIRI knowledge base**. Each resource in the knowledge base is associated with one or more functionalities. Functionalities are textual natural language descriptions, directly extracted from the papers. Each functionality is linked with one category and one domain of the existing resource list of categories and domains.

Figure 3 is a conceptual view of the knowledge base. As Figure 3 shows, each resource can be linked to one or more natural language descriptions of its functionalities--i.e. a tool may provide different functionalities. Each functionality consists of a natural language textual description extracted from the papers. The descriptions of the functionalities are connected with their respective categories and target domains. For instance, the 'FATCAT' resource in Figure 3 is linked to two different functionalities: 'Functionality 2.1' and 'Functionality 2.2'. Similarly, 'Functionality 2.1' is linked to the category 'Search' and to the domain 'Protein', whereas 'Functionality 2.2' is connected to the 'Comparison' category and to the 'Protein' domain. Therefore, the 'FATCAT' resource can be regarded as a tool for searching and comparing proteins. Similarly, 'CrossLink' can be identified as a tool for exploring RNA sequences, 'MATRAS' can be considered as a tool for aligning and comparing proteins and 'GenePublisher' can be identified as a resource for analyzing DNA microarrays.

## Results

We implemented the data extraction tool and the web services layer (WSL) providing the query primitives to access the knowledge base using the Java programming language and associated technologies [28]. This includes the Java Web Services Developer Pack (JWSDP) [29].

The system architecture includes a web services query layer developed to cover the contents of the knowledge base. To test the functionality of the developed WSL, we created a pilot web application on top of the WSL to provide users with a searchable web index of bioinformatics resources, by using popular web browsers including Internet Explorer^®^, Mozilla Firefox^® ^and Safari^®^.

**Figure 4:**
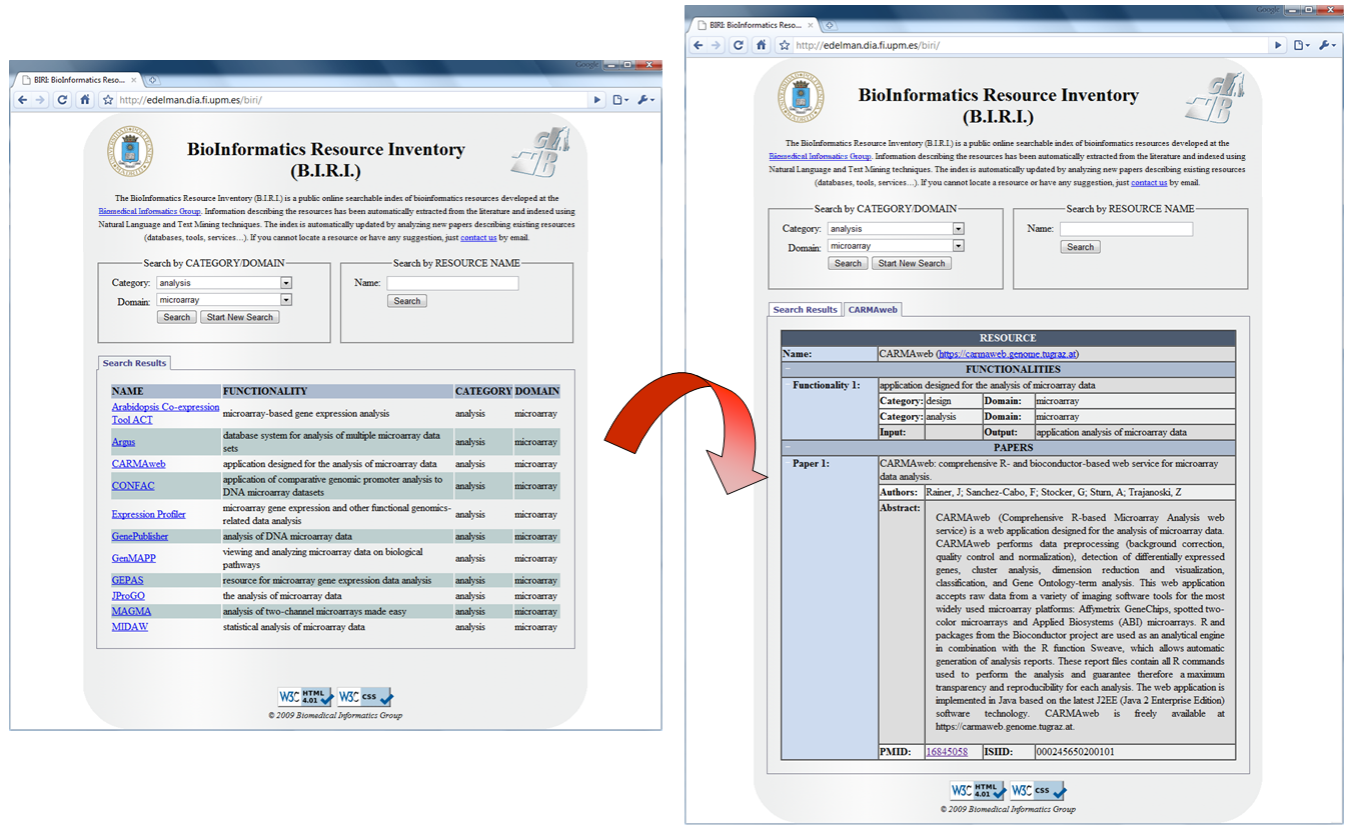
**Web Application Interface for Accessing the BIRI Knowledge Base**. The BIRI web application interface is useful for making search-by-category, search-by-domain and search-by-resource name queries. A list of resources satisfying a query is provided, showing the names of the resources plus links, functionalities, categories and domains.

As Figure 4 shows, the pilot web application provides the following search capabilities: i) retrieve all the information related to a specific resource given its name, and ii) search for relevant resources belonging to a given category and/or target domain. In both cases, the user is presented with a list of records matching the user query and containing basic information on the retrieved resources. This includes the resource name with a link to the complete information of the resource, a short textual description of the provided functionality, and its assigned categories and domains. Note that the indexing engine may have classified a single resource into multiple categories and domains, thus producing several entries in the results list. Clicking on the resource name, the system shows the complete information on this resource, including additional links to i) the official web page of the resource (if it is actually available in the abstract) and ii) the actual paper record at the PubMed^® ^website from which the information was extracted.

### A use case

Let us suppose that a researcher wants to identify resources for analyzing information from microarrays. The web application will show the existing categories and domains in two combo-boxes. These categories and domains are dynamically loaded using different web services. When the user selects a category, the domain combo-box is automatically updated to show the domains associated with the selected category only. Similarly, when the user selects a domain; the category combo-box is updated with the appropriate categories. For instance, the researcher may select "analysis" from the combo-box category. Then, the combo-box domain will be automatically updated with all the available domains for the chosen category. It includes an extra option for selecting "All" domains. The researcher will select the "microarray" domain from that list and then click on the Search button. The category and domain selection order is not mandatory. The user could select the domain first and the category afterwards.

After the user clicks the Search button, the web application displays a table with information about the resources contained in the knowledge base that meet the user-imposed restrictions. In the above example, the table contains eleven entries, as shown in Figure 4. Each entry contains the name of and a link to the resource, its functionality, category and domain. Further details on a given resource can be retrieved by clicking on the resource name link.

The web application has been tested with multiple queries selecting different combinations of categories/domains. Users could also search for a resource by its name. Searches by name are case-insensitive. The results would be similar but restricted to the specified resource.

Another interesting feature of the application is to incrementally update the index with new resources. This is achieved by entering the respective research papers in the update module. This module verifies the new papers added to prevent double entries. Papers that have been previously processed by the system are passed up, and the others are applied as described above. Users can also contribute by suggesting the inclusion of additional tools/abstracts by sending an email to the contact address provided on the BIRI web page.

### Evaluation

**Figure 5:**
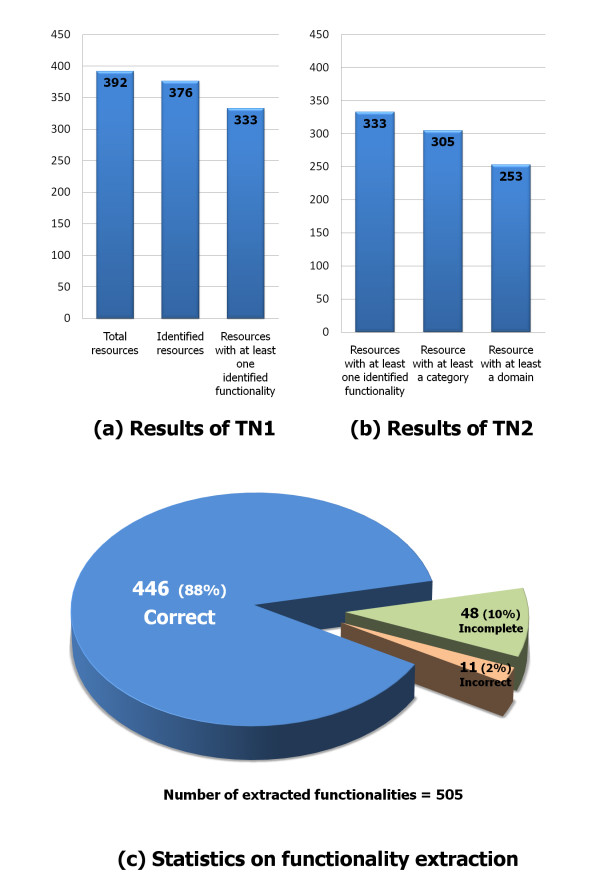
**Summary of experiment results**. The results of the experiment show that 88% of the functionalities were successfully extracted from the analyzed abstract. The functionality was incompletely extracted in 10% of the cases, and only 2% of the cases were incorrect.

As stated previously, resources are automatically classified and indexed according to a custom-created list of categories and domains shown in Table 2. This list is composed of 28 categories--i.e. resource types--targeting nine different domains--i.e. data types. We conducted an experiment using an input set of 400 abstracts extracted from the ISI Web of Knowledge^®^. To create the test set, we queried the system with the term "bioinformatics resources". Then, we sorted the results by impact factor and date, and finally we selected the first 392 documents (the most relevant) according to this classification [see Additional file 1]. We also included a small number of documents (eight, in this case) unrelated to bioinformatics resources to test the robustness of our approach. As Figure 5a shows, the first TN extracted the names of 376 resources (94%)--i.e. 24 manuscripts (6%) did not produce anything. From these 24 manuscripts, 16 papers (4%) were discarded even though they contained information about bioinformatics resources and the remaining 8 papers were the control set that we manually created and included for this experiment.

Regarding the functionality extraction process, the first TN discovered 505 descriptions of functionalities. They were assigned to the 333 identified resources. Note that a single resource may be assigned to one or more functionalities. As Figure 5c shows, a high percentage of the extracted functionalities (88%) provided complete and coherent descriptions. Conversely, 10% were incomplete descriptions that still provided valuable information for automatically extracting their associated category and domain. The remaining 2% matched up with incorrect or incoherent descriptions. The coherence of the extracted functionalities was manually assessed to verify the correctness of the extraction method.

Conversely, Figure 5b shows the results of the classification process carried out by the second TN. This was fed with the set of descriptions extracted by the first TN. The second TN successfully assigned at least one category to 305 resources, and at least one domain to 253 resources. Table 3 shows some sample entries of the generated knowledge base.

**Table 3:** Samples of short-form knowledge base entries

**Resource**	**Functionality**	**Category**	**Domain**
**CrossLink**	Exploration of relationships between RNA sequences	Exploration	RNA

**FATCAT**	Server for structure comparison and structure similarity searching	Comparison	Protein

**FATCAT**	Server for structure comparison and structure similarity searching	Comparison	Protein

**MATRAS**	Protein 3D structure comparison	Comparison	Protein

**MATRAS**	Accumulation structural employs progressive alignment algorithm 3D alignments useful tools for insights into protein 3D structures	Alignment	Protein

**GenePublisher**	Analysis of DNA microarray data	Analysis	DNA

**GenePublisher**	Analysis of DNA microarray data	Analysis	Microarray

The table shows examples of the knowledge base contents according with the resources shown in Figure 3. After executing a query, this is the information provided by the BIRI Web Application. It includes resource names and links, functionalities, categories, and domains.

These entries present the extracted information for four different bioinformatics resources. The first column shows the name of the extracted tools--i.e. "CrossLink", "FATCAT", "MATRAS" and "GenePublisher"--whereas the second column provides a short description of their functionality. The third column shows the category in which the corresponding resource has been classified. This classification is further refined in the fourth column, which lists the specific application domain for this resource. For instance, as shown in the Table 3, the "CrossLink" resource has been classified under the category "Exploration" and targets the "RNA" domain. Therefore, "CrossLink" can be regarded as a tool to explore RNA sequences. Also, additional information can be retrieved for each resource. Table 1 shows an example of all the information BIRI provides about any resource. This information has been automatically extracted from the scientific papers using the proposed method. It contains the resource name, functionalities, categories, domains, inputs, outputs and paper from which the information has been extracted. Links to the official web site of the resource and to the whole paper --in PubMed^®^-- are provided if they are available in the abstract.

To evaluate the suitability of our approach, we have compared BIRI with other existing public indexes according to two dimensions: i) features and characteristics provided by the indexes, and ii) the number of resources contained in each index that also appear in BIRI. Table 4 focuses on the first dimension--i.e. features and characteristics of the indexes. The information used to complete Table 4 has been extracted from the literature and the official web sites where the indexes are publicly available. We considered five different features: i) *generation of the index*, --i.e. whether the index was created manually or (semi-)automatically--, ii) *indexation of external resources*--i.e. whether other resources have also been indexed in addition to resources that actually belong to the institution or consortium that set up the index--, iii) the type of *search capabilities *provided by the index, iv) whether or not the *resources are annotated*, and v) whether the index includes any form of *resource classification*. As shown in Table 4, all the indexes that have been analyzed provide some kind of resource classification and annotation. Most of these indexes consider external resources and provide other advanced search capabilities. The major differences lie in the index generation process since, in most cases, the indexes are manually generated.

**Table 4:** Comparison of existing resource indexes

**NAME**	**Automatic Generation**	**External Resources**	**Advanced Search Capabilities**	**Annotation of Resources**	**Resources Classification**
***Bioinformatics Links Directory ***[6,7]		X	X	X	X

***Pathguide ***[37]		X	X	X	X

***Online Bioinformatics Resource Collection ***[38]		X	X	X	X

***ExPASy Life Science Directory ***[39]		X		X	X

***Molecular Biology Database ***[5]		X		X	X

***Database of Databases ***[40]		X	X	X	X

***Resources at the EBI ***[8]			X	X	X

***iTools ***[17,18]	X	X	X	X	X

***myGrid ***[41]		X		X	X

***Feta ***[42,43]	Semi-automatic	X	X	X	X

***caBIG ***[44]			X	X	X

**BIRI**	X	X	X	X	X

We compare existing indexes containing information about resources. We considered several classification criteria: i) automatic index generation, ii) whether or not the index indexes external resources, iii) whether the index interface provides advanced search capabilities, iv) whether or not the resources are annotated, and v) whether the index establishes a resources classification.

Table 5 deals with the second dimension of the evaluation. It compares the indexes according to the number of resources that they contain. The yardstick was the curated BIRI knowledge base. After the curation process, it contained 316 different resource names. Then, we compared BIRI with the other indexes listed in Table 4. From this comparison, we were able to obtain the full list of stored resources. Table 5 shows the total number of resources for each index, the number of matches with BIRI--i.e. the number of resources that appear simultaneously in both indexes--, and the number of resources discovered by BIRI that the other indexes do not contain. As we can see in the table, BIRI obtains high matching rates (over 73%) with two indexes--the BLD and the Online Bioinformatics Resource Collection. Matching rates for the other indexes are from 3% to 10%. The difference is due to the kind of resources each index contains. The indexes containing heterogeneous resources, such as BIRI, achieve higher matching rates than others containing only one type of resources, such as Molecular Biology Database or Database of Databases --which only index databases.

**Table 5:** Evaluation of the BIRI contents against other indexes

**Index Name**	**Total Resources Indexed**	**Matches**	**New in BIRI**
***Bioinformatics Links Directory ***[6,7]	1350	235	81

***Pathguide ***[37]	293	10	306

***Online Bioinformatics Resource Collection ***[38]	2368	231	85

***ExPASy Life Science Directory ***[39]	1253	15	301

***Molecular Biology Database ***[5]	1171	28	288

***Database of Databases ***[40]	1056	33	283

***Resources at the EBI ***[8]	111	22	294

We compare the resources contained in the curated BIRI knowledge base with the resources indexed by other public indexes at the time of writing this paper. The table shows: i) the total number of resources indexed by each index, ii) the number of matches found between BIRI and the index, and iii) the number of resources existing in BIRI knowledge base that do not exist in the other index.

## Discussion

Based on pattern matching methods, our method can automatically create a knowledge base of bioinformatics resources by i) detecting resource names, ii) extracting short descriptions of functionalities and iii) classifying the extracted artefacts according to a list of categories and domains of bioinformatics resources, which extends the BLD classification [7] on which our list is based. We believe that creating a standardized taxonomy or ontology of bioinformatics resources is a crucial task to facilitate collaborative and integrative approaches [1].

For the methodology proposed in this paper to work, the bioinformatics resources have to have been previously published and indexed in PubMed^® ^or the ISI Web of Knowledge^®^. Otherwise, the resources would never be found using this method. In contrast, our approach guarantees that only high-quality resources are indexed. Once these resources have passed a peer-review process, confidence in their quality can be actually higher.

Table 4 compares our approach with the most relevant indexes available at the time of writing this paper --and considering that BIRI is a prototype, currently being expanded. As shown in Table 5, from an original set of over 400 papers, the system automatically discovered more than 230 resources that also appear in BLD or the Online Bioinformatics Resource Collection. Another interesting fact pointed out by Table 5 is that BIRI contains several resources not included in other indexes. The number of new resources in BIRI ranges from 81 (when compared to BLD) to 306 (when compared to Pathguide). This happens since existing indexes are often updated only considering manuscripts published in a reduced set of journals. This limited vision hinders the creation and maintenance of an exhaustive list of resources. Conversely, our methodology is not centered in any particular journal. It follows a more general approach using PubMed^® ^or the ISI Web of Knowledge^® ^as information sources. This provides us with a broader vision of recent developments. Once new manuscripts are available in PubMed^® ^or the ISI Web of Knowledge^®^, our system can be updated with the new resources.

Our approach extends most of the search capabilities provided by other existing tools. In addition, the index is automatically generated and updated in our approach. The update process is a very time-consuming and tedious task that is usually performed manually by groups of experts. Applying the same methodology detailed above, the contents of our index and knowledge base can be automatically updated by just entering new manuscripts and abstracts into the tool.

Our index provides users with advanced search capabilities. It can, for instance, perform complex searches, such as searching for resources matching a definite category and/or target domain. As stated previously, our knowledge base is built automatically using pattern-matching techniques, whereas other indexes are created, maintained and updated manually. Besides, our knowledge base can be automatically updated with new resources simply by feeding the developed information extraction tool with manuscripts describing recently developed tools, databases and services.

Considering other existing indexes, our knowledge base provides additional information, such as, for instance, the target domains where the different types of resources can be applied or the resource inputs and outputs. Using the resources' names and extending the information about inputs and outputs, BIRI could be useful for automatically orchestrating workflows like applications combining several resources. An example of resource combination through workflow definition is described in a previous work carried out by the authors, where multiple databases are queried to retrieve information regarding the proteins involved in a genetic disease [30]. The results provided by a database are used to build the query for the next database.

Additionally, text-mining based methods for information extraction reported that they could benefit from manuscripts with more structured abstracts [31].

## Conclusion

Our tool automatically extracts and organizes relevant information about bioinformatics resources from research papers describing the resources. Our method, based on a domain-independent approach, can be used to create inventories targeting different scientific fields [32,33]. For instance, this approach is currently being applied in the European Commission-funded Action-GRID project, coordinated by the authors [34]. In one of the workpackages of this project, we are creating an inventory of bioinformatics and nanomedical resources that is intended to help researchers in these areas [35].

Several initiatives have been carried out aimed at cataloguing the existing bioinformatics resources. Although our tool could work as a standalone application, it has not been designed for this purpose. Our tool is intended not as a replacement for but an add-on to existing indexes and applications. We are working on integrating our tool as a plug-in for other consolidated applications, such as BioPortal or BioMoby. Additionally, tools for defining workflows, such as TAVERNA [36], could also benefit from the information provided by an extended version of our index.

The inventory of resources could also be collaboratively updated by other external contributing researchers. This collaborative approach is being successfully applied in other fields, such as, for instance, developing biomedical ontologies at the NCBO [3]. At the NCBO, different collaborative tools--developed using Web 2.0 techniques--are available for developing biomedical ontologies. Researchers can contribute by entering new information or comments about the existing information.

## Authors' contributions

GDLC conceived and participated in the design of the study and drafted the manuscript. MGR conceived and participated in the design of the study and drafted the manuscript. SC participated in the design of the study and the evaluation of the results. DDLI participated in the design of the study and implemented the system. VM conceived the study and helped to draft the manuscript. All authors read and approved the final manuscript.

## Supplementary Material

Additional file 1 **PubMed identifiers list**. This file contains the list of the 392 PubMed identifiers selected to create the test set. Click here for file
